# Cardiac Manifestations of Myotonic Dystrophy in a Pediatric Cohort

**DOI:** 10.3389/fped.2022.910660

**Published:** 2022-06-09

**Authors:** Laia Brunet Garcia, Ankita Hajra, Ella Field, Joseph Wacher, Helen Walsh, Gabrielle Norrish, Adnan Manzur, Francesco Muntoni, Pinki Munot, Stephanie Robb, Rosaline Quinlivan, Mariacristina Scoto, Giovanni Baranello, Anna Sarkozy, Luke Starling, Juan Pablo Kaski, Elena Cervi

**Affiliations:** ^1^Hospital de Mataró, Barcelona, Spain; ^2^Great Ormond Street Hospital Children's Charity, London, United Kingdom

**Keywords:** myotonic dystrophy (DM1), congenital myotonic dystrophy, pediatric population, neuromuscular disorder, cardiac conduction disease, electrocardiographic abnormalities

## Abstract

Myotonic dystrophy type 1 (DM1) is the most prevalent inherited neuromuscular dystrophy in adults. It is a multisystem disease with cardiac manifestations. Whilst these are well-defined in adults, there are scarce published data in the pediatric population. This study aimed to investigate the yield and progression of cardiac disease in pediatric DM1 patients, focusing on congenital DM1 (cDM1).

**Methods:**

A retrospective observational study of all pediatric DM1 patients referred to our center (December 2000-November 2020) was conducted. Patients were classified into DM1 forms according to age of symptom onset and disease severity. Patients underwent clinical and cardiac evaluation with 12-lead ECG, transthoracic echocardiography and 24-h ECG Holter monitoring.

**Results:**

67 DM1 pediatric patients were included: 56 (83.6%) cDM1 and 11 (16.4%) non-cDM1. Median follow-up time of cDM1 patients was 8.0 [3.25–11.0] years. 49 (87.5%) cDM1 patients had baseline 12-lead ECG and 44 (78.6%) had a follow-up 12-lead-ECG, with a median follow-up time from diagnosis to baseline ECG of 2.8 [1.0–8.5] years and to follow-up ECG of 10.9 [5.7–14.2] years. Overall, 43 (87.8%) presented ECG abnormalities, most commonly in the form of asymptomatic conduction disease (*n* = 23, 46.9%), of which 21 (42.9%) had first degree atrioventricular block (1^st^ AVB). There was an increase of prevalence from baseline to follow-up ECG in low QRS voltage (16.7%), poor R wave progression (13.9%), abnormal repolarisation (11.9%) and 1^st^ AVB (7.6%). one patient (1.8%) underwent pacemaker implantation for syncope in the context of progressive conduction disease. No patients developed left ventricular systolic dysfunction. 4 (7.1%) cDM1 patients died during follow up, including three who died suddenly with no clear cause of death.

**Conclusions:**

This study is the first to analyse the prevalence and progression of ECG abnormalities in cDM1 pediatric patients. The high prevalence of abnormal findings, progressive changes and number of potentially associated events (1 pacemaker implantation and 3 unexplained sudden deaths) stresses the importance of systematic and continued cardiac evaluation of these patients.

## Introduction

Myotonic dystrophy type 1 (DM1) is the most prevalent inherited neuromuscular disease in adults with 1:8000 incidence (1). It is caused by an autosomal-dominant expansion of a cytosine–thymine–guanine (CTG) trinucleotide repeat on chromosome 19q13.3 (2). Anticipation in consecutive generations associates with earlier onset and an increased severity of the disorder (3). This multisystem disease is primarily characterized by progressive muscle weakness and myotonia, but can include endocrine, respiratory, central nervous, gastrointestinal, ocular, urinary and cardiac manifestations. The risk of sudden death in DM1 patients has been reported to be 0.56% per year (4). Based on age of onset and the clinical severity, pediatric DM1 patients can be divided into congenital (cDM1), infantile (iDM1) and juvenile (jDM1) forms (4). cDM1 patients is the more severe form with the lowest life expectancy (5). The mortality rate is up to 40% in the neonatal period due to respiratory diseases and the mean life expectancy is 45 years (6).

cDM1 presents with hypotonia at birth, respiratory failure, difficulties with feeding and developmental delay. cDM1 is almost exclusively associated with maternal inheritance (7).

Cardiac manifestations in adults with DM1 include ventricular dysfunction, progressive conduction defects and ventricular arrhythmias which can lead to sudden cardiac death (SCD) (1). Nonetheless, data on progressive cardiac abnormalities in the pediatric population are scarce. This study, therefore, aimed to investigate the prevalence, progression and clinical impact of cardiac disease in pediatric DM1 patients, focusing on cDM1.

## Methods

### Data Collection

A retrospective observational study was conducted of all consecutive pediatric individuals (aged ≤ 18 years) with a diagnosis of DM1 seen at Great Ormond Street Hospital between December 2000 and November 2020. The study was approved by the Research Board and consent waived in view of the retrospective data collection. Electronic patient records were systematically reviewed.

### Clinical Evaluation

DM1 was clinically diagnosed and confirmed by genetic testing. The following clinical forms were defined owing to the age of onset of first clinical manifestation: cDM1 (birth-1 month), iDM1 (1 month—10 years) and jDM1 (11-20 years) (4).

We specifically developed a systemic severity score that consisted of the 8 more representative extra-cardiac features from our cDM1 cohort: learning difficulties, non-invasive ventilation, fecal incontinence, nasogastric or gastrostomy feeding, dysphagia, sleep disorder, urinary incontinence and full/partial wheelchair dependence. This score was evaluated in each patient at the end of the study period.

Patients underwent annual cardiac evaluation including medical and family history, physical examination, resting 12-lead ECG; transthoracic echocardiography; signal averaged ECG (SAECG); and ambulatory 24-h ECG Holter monitoring (AECG). Nevertheless, if cardiac symptoms and/or significant ECG abnormalities were observed, patients were more frequently evaluated.

Exercise testing (ETT) is recommended for young DM1 patients as physical exertion has been reported to be pro-arrhythmogenic (8, 9). However, we performed an ETT depending on the age range and physical capability of our patients. We used a treadmill test following a modified Bruce protocol. Electrophysiological study (EPS) was carried out when clinically indicated according to current guidelines to check for conduction abnormalities not apparent on surface ECG as per standard practice. Specifically, it was performed for establishment of atrioventricular block as the main cause of symptoms, and for identification of the anatomic site of block that may dictate the potential need of permanent pacing (8, 10, 11). Clinical data were collected at baseline and during follow-up until patients were transitioned to adult services around the age of 18, the end of the study period or until patient's death.

### Electrocardiographic Analysis and Interpretation Criteria

12-lead ECG was recorded at a paper speed of 25 mm/s and amplitude of 10 mm/mV. When more than one tracing was available, baseline (ECG1) and latest available follow-up 12-lead ECG (ECG2) were analyzed independently and blindly by two investigators (LB and EC), and re-reviewed by the senior author (EC) when assessor opinion varied. Normal limits for ECG parameters were defined according to Rijnbeek et al. and current guidelines (12–14).

We defined sinus bradycardia as a heart rate ≤2^nd^ percentile (12, 13). Mean frontal plane electrical axis was considered normal between 0° and 120° (birth-1 month); between 0° to 90° (1 month-16 years) and between −30° and 90° (>16 years). We defined left-axis deviation when the mean frontal plane electrical axis was <0° (birth-16 years) or <-30° (>16 years); right-axis deviation from 120° to 180° (in the first month of life) and from 90° to 180° (adults); extreme-right axis deviation between −90° and 180°. The axis was considered as indeterminate when there were isodiphasic QRS complexes in the frontal plane, with no dominant QRS deflection (14). Poor R wave progression was established when R wave amplitude in lead V3 was ≤3 mm and R wave amplitude in lead V2 was ≤ to the R wave amplitude in V3 (15). Low QRS voltage was defined as QRS amplitude ≤5 mm (0.5 mV) in each peripheral lead and/or QRS amplitude ≤10 mm (1 mV) in each precordial lead (16). Non-specific intraventricular conduction delay was defined according to current guidelines (14). SAECG was recorded at 40 Hz high-pass filtering. The presence of late potentials was determined in patients with a QRS <110 ms considering 3 SAECG parameters: filtered QRS duration ≥114 ms, duration of terminal QRS <40 μV or a root-mean-square voltage of the last 40 ms of QRS <20 μV (17). We considered SAECG to be abnormal when ≥2 parameters were abnormal. Conduction defect was defined as the presence of first or higher degree atrioventricular block (AVB), left or right bundle branch block (RBBB) or left anterior fascicular block (LAFB) on 12-lead ECG (14). ECG was considered abnormal when any of the aforementioned alterations and/or abnormal repolarisation (considering T wave abnormalities and QTc duration) were present (18). AECG was considered to be abnormal when any of the previously described ECG abnormalities were found.

### Cardiac Imaging

Echocardiogram data were collected during follow-up. Cardiac dimensions were assessed against normal values as per recent published datasets (19). Standard clinical parameters were used to define structural cardiac abnormalities. Normal left ventricular systolic function was defined as left ventricular ejection fraction >55% (20).

### Statistics

Normally distributed data are presented as mean values [±standard deviation (SD)] and non-normally distributed variables as a median [interquartile range (IQR)]. Categorical variables are presented as number (n) and percentages (%).

We explored for normality by a Kolmogorov-Smirnov test. Chi-squared or Fisher's exact test were used for comparing of categorical variables and Student's *t*-test for continuous measurements. A *P* < 0.05 was considered to be statistically significant for all data. Significance was only analyzed where n≥3 in each group. The variant “learning difficulties” was not included in the statistical analysis as it was present in all cDM1 patients. Statistical analysis was performed using R Studio software version 1.2.1335.

## Results

The study cohort consisted of 67 DM1 pediatric patients: 56 (83.6%) cDM1, 8 (11.9%) iDM1 and 3 (4.5%) jDM1. Table 1 shows baseline demographic characteristics of DM1 patients; Supplementary Table 1 the non-cardiac clinical manifestations. Due to the small number of non-cDM1 patients and potential for a different prevalence of cardiovascular findings in the different subgroups, we divided the cohort into cDM1 and non-cDM1. Results are presented separately.

**Table 1:** Baseline characteristics of patients with DM1.

**Baseline information**	**cDM1** **(*n* = 56)**	**iDM1 and jDM1** **(*n* = 11)**
Female, *n* (%)	33 (58.9)	7 (63.6)
Gestational age		
Pre-term, *n* (%)	27/50 (54.0)	1/10 (10.0)
Mean gestational age pre-term patients (±SD)	33.9 (±2.5)	36 (±0)
Polyhydramnios, *n* (%)	23/31 (74.2)	0/6 (0)
Mean age at first symptoms, months (±SD)	0.01 (±0.02)	100 (±66.5)
Mean age at genetic diagnosis, years (±SD)	1.4 (±2.9)	8.5 (±5.7)
Ethnicity		
Caucasian, *n* (%)	40 (71.4)	9 (81.8)
Asian, *n* (%)	7 (12.5)	0 (0)
Other/not known, *n* (%)	5 (8.9)	2 (18.2)
Maternally inherited, *n* (%)	54 (96.4)	3 (27.3)
Paternally inherited, *n* (%)	2 (3.6)	8 (72.7)
Median follow-up, years [IQR]	8.0 [3.3–11.0]	3.0 [1.0–11.0]
Symptoms (palpitations, syncope, chest pain or dizziness)		
Absent, *n* (%)	49 (87.5)	8 (81.8)
Palpitations, *n* (%)	2 (3.6)	0 (0)
Syncope, *n* (%)	2 (3.6)	1 (9.1)
Chest pain, *n* (%)	2 (3.6)	0 (0)
Dizziness, *n* (%)	1 (1.8)	0 (0)
Pacemaker, *n* (%)	1 (1.8)	0 (0)
Implantable cardioverter defibrillator, *n* (%)	0 (0)	0 (0)

*Fractions give the absolute number of patients divided by the number of patients with available clinical information for each item. Values are n (%)*.

*cDM1, Congenital Myotonic Dystrophy type 1; iDM1, Infantile Myotonic Dystrophy type 1; jDM1, Juvenile Myotonic Dystrophy type 1; IQR, Interquartile Range; SD, Standard Deviation*.

### Congenital Myotonic Dystrophy Type 1 Cohort

#### Clinical Events and Symptoms

During follow-up, 7 (12.5%) cDM1 patients experienced symptoms (Table 1). One patient presented at 8 years of age with 2 syncopal episodes with clonic movements and prolonged post-ictal period which were considered seizures. At the age of 11, he had recurrence of syncope and investigations documented progressive atrioventricular conduction defects on 12-lead ECG and AECG in the form of 1^st^ AVB, second degree AVB Mobitz type I, Mobitz type II and 2:1 AVB. His AECG additionally showed a median heart rate of 84 bpm (minimum of 56 bpm and maximum of 141 bpm) with no significant sinus pauses nor arrhythmias. Due to his syncopal episode in the context of second degree AVB, according to international guidelines, he underwent an elective EPS which confirmed conduction disease (HV interval of 60 ms) with no inducible sustained ventricular tachycardia (8). Therefore, a transvenous pacemaker was implanted, according to current guidelines (Class I recommendation) (11). This patient is alive and symptom-free, with no ventricular arrhythmias documented through pacemaker downloads and has not required further interventions after 32 months of follow-up. No patients underwent implantable cardioverter defibrillator implantation.

Four (7.1%) patients died at a median age of 8.5 [6.25-16.75] years. One of them (1.8%) died due to progression of the disease and respiratory failure; he had right axis deviation, low QRS voltage and abnormal repolarisation at both ECG1 and ECG2. The remaining deaths (*n* = 3, 5.4%) were sudden and unexplained, with no available data on death circumstances and no post-mortem examination performed. The ECG was only available for two out of these three patients, showing right axis deviation and indeterminate axis, low QRS voltage and abnormal repolarisation. One developed 1st AVB and non-specific intraventricular conduction delay at follow-up. They were asymptomatic.

Supplementary Table 2 shows the comparison of ECG abnormalities in the deceased and alive patients with cDM1; Supplementary Table 3 shows the association between mortality and the presence of systemic features of cDM1. Overall, there was a trend toward statistical significance between mortality and a higher systemic severity score (*P* = 0.088).

Supplementary Figure 1 depicts the number of systemic features in cDM1 patients as a surrogate for clinical severity. Median severity score was 7.5 [4.75–8.0] in deceased patients and 4.0 [3.0–6.0] in alive patients.

#### Cardiac Investigations

Diagnostic work-up is shown in Supplementary Figure 2.

##### 12-Lead ECG and SAECG Findings

Forty-nine patients (87.5%) had an ECG1 and 44 (78.6%) at least one ECG2, with median interval of 7.5 [5.2–5.6] years between the two (Table 2). Supplementary Figure 3 depicts QRS and PR interval distribution at ECG1 and ECG2 (12–14).

**Table 2:** Electrocardiographic findings of congenital DM1 patients.

**Baseline information**	**Baseline ECG** **(*n* = 49)**	**Follow-up ECG (*n* = 44)**	***P*** **-value**	**Increased prevalence (%)**
Median age at the ECG, years [IQR]	4.1 [1.7–10.5]	12.2 [6.9–16.1]		
Sinus bradycardia	1 (2.0)	1 (2.3)		0.3
1st degree AV block, *n* (%)	13 (26.5)	15 (34.1)	0.5465	7.6
Nonspecific intraventricular conduction delay	12 (24.5)	13 (29.6)	0.3711	5.1
Right BBB, *n* (%)	2 (4.1)	0 (0)		
Left BBB, *n* (%)	0 (0)	0 (0)		
Left Anterior Fascicular Block, *n* (%)	2 (4.1)	5 (11.4)		7.3
QRS axis				
Normal, *n* (%)	21 (42.9)	18 (40.9)	>0.999	
Left axis deviation, *n* (%)	7 (14.3)	10 (22.7)	0.1824	8.4
Right axis deviation, *n* (%)	12 (24.5)	9 (20.5)	0.505	
Superior axis deviation, *n* (%)	5 (10.2)	5 (11.4)	>0.999	1.2
Indeterminate axis, *n* (%)	6 (12.2)	4 (9.1)	0.6171	
Low QRS voltage, *n* (%)	13 (26.5)	19 (43.2)	0.023	16.7
Poor R-wave progression, *n* (%)	1 (2.0)	7 (15.9)		13.9
Abnormal repolarisation (Flat/inverted T waves)	12 (24.5)	16 (36.4)	0.4227	11.9
Inferiorly	4 (8.2)	4 (9.1)	>0.999	0.9
Inferolaterally	3 (6.1)	8 (18.2)	0.2278	12.1
Inferior and anterior leads	1 (2.0)	1 (2.3)		0.3
Generalized	4 (8.2)	3 (6.8)	>0.999	
QTc ≥450	1 (2.0)	1 (2.3)		0.3
Overall ECG abnormalities	43 (87.8)	43 (97.7)	0.1336	9.9

*AV, Atrioventricular; BBB, Bundle Branch Block; DM1, Myotonic Dystrophy type 1*.

Overall, forty-three of the forty-nine patients (87.8%) that underwent at least one ECG had ECG abnormalities: abnormal repolarisation (*n* = 21, 42.9%), 1^st^ AVB (*n* = 21, 42.9%), low QRS voltage (*n* = 19, 38.8%), non-specific intraventricular conduction delay (*n* = 17, 34.7%) and poor R wave progression (*n* = 9, 18.4%).

Twenty-six (44.8%) patients had a SAECG (Supplementary Table 4).

##### Ambulatory 24-h ECG Holter Monitoring and Exercise ECG Findings

Forty patients (71.4%) had an AECG. No patients had significant arrhythmias or sinus pauses (>3 s), 14 (35.0%) had occasional isolated supraventricular ectopics, 11 (27.5%) isolated ventricular ectopics and 3 (7.5%) junctional rhythm. Table 3 includes conduction defects after 12-lead and AECG analysis.

**Table 3:** Overall conduction defects in congenital DM1 patients with at least one 12-lead ECG including 12-lead ECG and ambulatory ECG monitoring data.

**Conduction defects**	**Study population** **(*n* = 49)**
Any conduction defect, *n* (%)	23 (46.9)
1st degree AV block, *n* (%)	21 (42.9)
Isolated 1st degree AV block, *n* (%)	13 (26.5)
And RBBB *n* (%)	1 (2.0)
And Left anterior fascicular block, *n* (%)	4 (8.2)
And RBBB and Left anterior fascicular block, *n* (%)	0 (0)
And Mobitz type I and II and 2:1 AV block, *n* (%)	1 (2.0)
Isolated left anterior fascicular block, *n* (%)	1 (2.0)
Isolated RBBB, *n* (%)	1 (2.0)

*AV, atrioventricular; RBBB, Right Bundle Branch Block*.

Two of the three patients (5.4% of the total cohort) who underwent an ETT had 1st AVB at ECG1. There was no evidence of any higher degree block on exercise. All three patients had sinus rhythm with no arrhythmias on exertion and were asymptomatic throughout the test.

##### Association Between ECG Findings and Extracardiac Features

There was no statistical association between the presence of any conduction defect and systemic features of cDM1 (Supplementary Table 5) nor with the use of invasive ventilation in neonatal period (*P* = 0.246) or a significant difference with a higher median systemic severity score (*P* = 0.587). There was a statistically significant difference between abnormal SAECG and a greater systemic severity score (*P* = 0.029). There was no significant difference between median systemic severity score and an abnormal ECG (Supplementary Table 6).

There was a statistically significant association between low QRS voltage at ECG1 and low QRS voltage at either ECG1 or ECG2 and the need for non-invasive ventilation in the neonatal period (*P* = 0.022 and *P* = 0.017, respectively). Nevertheless, there was no significant association between the latter and low QRS voltage at ECG1 (*P* = 0.338), and abnormal repolarisation at ECG1 or ECG2 (*P* > 0.999 and *P* = 0.365, respectively).

##### Cardiac Imaging

Data regarding transthoracic echocardiography was available for 54 patients (96.4%) (Table 4). One (1.9%) developed asymmetric hypertrophy (HCM), mild mitral valve prolapse (MVP) and mild ascending aortic dilatation; additional genetic tests identified a variant of unknown significance in *TPM1* and *KCNQ1* genes. Five premature patients (9.3%) (median gestational age 34 [29.6–36.1] weeks), had a haemodynamically significant patent ductus arteriosus (PDA). No patients developed dilated cardiomyopathy or left ventricular systolic dysfunction.

**Table 4:** Echocardiography data for congenital DM1 patients.

**Echocardiography features**	**Study population (*n* = 54)**
Left ventricular ejection fraction, % [IQR]	68.5 [62.5–72.0]
Left ventricular dimensions, z-score [IQR]	−1,8 [−0, 1–0, 0]
Echocardiographic abnormalities (total)	8 (14.8)
Pericardial effusion without haemodynamic compromise, *n* (%)	1 (1.9)
PDA (surgical closure), *n* (%)	3 (5.5)
PDA, VSD and left lower pulmonary vein stenosis, n (%)	1 (1.9)
Isolated PDA	2 (3.7)
PDA (interventional closure), *n* (%)	2 (3.7)
PDA and mild aortic root dilatation, *n* (%)	1 (1.9)
Isolated PDA	1 (1.9)
HCM, mild MVP and Aortic root dilatation, *n* (%)	1 (1.9)
Left aortic arch with right aberrant subclavian artery	1 (1.9)

*HCM, Hypertrophic Cardiomyopathy; MVP, Mitral Valve Prolapse; PDA, Patent Ductus Arteriosus; VSD, Ventricular Septal Defect*.

### Non-congenital Myotonic Dystrophy Type 1 Cohort

Table 1 contains baseline characteristics of patients with iDM1 and jDM1.

Nine (81.8%) non-cDM1 patients had an ECG1 which was abnormal in 6 (66.7%): 1st AVB and RBBB (*n* = 1, 16.7%), 1st AVB (*n* = 1, 16.7%), low QRS voltage and non-specific intraventricular conduction delay (*n* = 2, 33.3%), non-specific intraventricular conduction delay (*n* = 1, 16.7%), and non-specific intraventricular conduction delay, low QRS voltage and left axis deviation (*n* = 1, 16.7%). Two of the six patients (33.3%) that had an ECG2 had non-specific intraventricular conduction delay. One iDM1 patient (9.1%) had a small perimembranous ventricular septal defect and another an insignificant interatrial septal defect, both with no haemodynamic compromise.

## Discussion

To the best of our knowledge, this is the largest study so far evaluating cardiac involvement and its progression in an exclusively pediatric cDM1 cohort. We have observed a high yield of electrocardiographic abnormalities and progression throughout follow-up in pediatric cDM1 patients, which has not been previously reported. Additionally, we demonstrated a potential risk of SCD at a young age among cDM1 patients.

### Conduction Defects

Our analysis also demonstrates a high incidence of ECG abnormalities in cDM1 patients compared to a previous study by Ho et al. including pediatric DM1 patients (7). A potential explanation for this discrepancy could be that patients with cDM1 are more severely and prematurely affected compared with patients with milder forms of DM1 (non-cDM1).

Particularly, among ECG abnormalities, the major concern was related to conduction defects. Although we demonstrated progression of conduction defects, high degree conduction disease was rare with only one patient requiring pacing. We did not routinely perform EPS in our young patients in view of its invasive nature but we used it to assess conduction properties and confirm indication for pacing in a patient in the context of syncope and second degree AVB, according to international guidelines (Class I recommendation) (8).

First AVB has previously been reported as the most common conduction defect in adult DM1 patients (1, 21). Nevertheless, our data evidences a higher prevalence of 1^st^ AVB in pediatric DM1 patients than adults and than a previous study by Sharma et al, from a cohort of pediatric cDM1 patients (22). This highlights the early conduction defects manifestations that cDM1 patients have, possibly in line with the more severe clinical expression described in the congenital onset compared to the other clinical forms. Moreover, the predominance of maternal inheritance in our cDM1 cohort reinforces the concept of an earlier and more severe phenotype from the systemic perspective and it can be speculated that cardiac abnormalities follow a similar pattern (3). Additionally, consistently with previous reports, in our cohort, 82.6% of the patients with a conduction defect were asymptomatic (23).

### ECG Findings and Extracardiac Features

In this study, low QRS voltage and abnormal repolarisation were the first and second more frequent ECG abnormality at ECG2, respectively. This could be partially explained by long standing lung pathology and chest deformities of these patients as we found a statistically significant association between low QRS voltage at ECG2 and the need for non-invasive ventilation in the neonatal period. However, there was no significant association between the latter and the presence of abnormal repolarisation at ECG2. Nevertheless, due to its high prevalence in our study, these findings might be considered as an expression of myocardial involvement in these patients as diseases progresses. In this regard, there was a statistically significant difference between low QRS voltage prevalence at ECG2 compared to ECG1. Moreover, right-axis deviation and indeterminate axis were more frequent at ECG1 than at ECG2. This could be related to a higher prevalence of prematurity and need for ventilator support in cDM1 with possible electrical findings reflecting higher pulmonary pressures and lung pathology, with subsequent right heart involvement. This would be in keeping with, at least, partial regression of the findings as they got older and were weaned from support.

In our study, there was a trend toward a statistical significance between low QRS voltage and increased severity score, and low QRS voltage and mortality approached statistical significance. Additionally, the median severity assessed through systemic score was higher in deceased patients, which would be in keeping with the significance between low QRS voltage and severity score and mortality. Low QRS voltage had previously been associated with a more severe cardiac involvement in amyloidosis but this has not been proven in cDM1 patients (16). Low QRS voltage has also been reported associated with an increased risk of mortality in individuals apparently free of cardiovascular disease (16, 24). Further studies including larger sample sizes are warranted to confirm our findings.

### Arrhythmias

Although arrhythmias are known to be the major cardiac manifestations of DM1, we did not document significant supraventricular or ventricular arrhythmias. Despite systematic ETT being recommended as Class IIB indication, we could only exercise a small number of patients due to their muscular impairment and inability to exercise with disease progression (8). Physical exercise has been previously reported to be an arrhythmogenic factor and it remains to be determined if the risk of malignant arrhythmia is driven by adrenergic stimuli and how that impacts a population that has a very low level of exercise (9). Another contributing factor could have been the lower age of our cohort, as arrhythmias are age-dependent in DM1 population, reported as occurring in the second decade of life (9).

### Sudden Death

PR ≥ 200 ms, QRS ≥ 120 ms and QTc ≥ 450 ms have been described to be predictors of SCD in adult DM1 patients (21). In our cohort, none of the patients that died fulfilled these adult criteria. Complete heart block leading to asystole or ventricular arrhythmias had been documented as potential mechanisms leading to SCD (8). In our study, 3 out of the 4 deaths were sudden and unexpected, and an arrhythmia could not be ruled out as a cause of the death although mild ECG abnormalities were previously documented. Interestingly, all of them had low QRS voltage. Their deaths were unexplained as no autopsy was performed. Nevertheless, they raise concerns about the potential risk of SCD at a young age in this population given the small sample size and number of events observed.

### Additional Cardiac Findings

In this study, the patient with HCM diagnosis was also heterozygous for a variant of uncertain significance in TPM1 and in KCNQ1 genes. Despite HCM having been rarely reported associated with DM1, it is difficult to determine the contribution of these two variants in his cardiac phenotype in the context of cDM1 diagnosis (5). Even if not common, an association between DM1 and dilated cardiomyopathy and heart failure has been reported later in life. None of our patients developed these two features, probably due to their age related penetrance in DM1 patients (8). MVP has been reported in 25-40% conversely to our findings, where 1.9% had MVP. Moreover, in our study, 9.3% of the patients that underwent echocardiography had a haemodynamically significant PDA. Although this finding has not been previously reported in DM1 patients, this is most likely due to the younger patients included and that all of them were ex-preterm babies.

### Clinical Implications of Our Results

Our finding of a high prevalence of cardiac abnormalities and its progression throughout childhood in cDM1 patients stresses the vital importance of their regular clinical assessment, particularly to monitor the development of conduction defects as, together with ventricular arrhythmias, place DM1 patients at a higher risk of SCD (8). According to current guidelines, cardiac evaluation including examination, 12-lead ECG, echocardiogram and AECG monitoring at baseline are recommended as a Class IC indication, even in asymptomatic patients (8). Further follow-up with the same investigations are recommended as a Class IIa indication in patients with normal cardiac investigations at baseline (3, 8, 11). Our results support the validity of the investigations proposed by current guidelines as we have demonstrated that the ECG is almost invariably abnormal, there is a potential for progression and higher degrees of AVB were recorded during AECG. The high prevalence of clinical events including syncope due to AV conduction disease (1/49, 2%) and unexplained deaths (3/49, 6.1%) stresses how screening is paramount and better risk stratification is still needed. We would recommend performing the same initial clinical evaluations as current guidelines with the addition of SAECG. We would suggest annual follow-up with the same investigations unless a progression or higher degree of AVB is observed. Performing an ETT will depend on the age range and/or physical capability of these patients.

## Limitations

The study is limited by the small cohort of patients, probably due to the fact that cDM1 is a rare disease. Not all patients had an available baseline ECG nor a follow-up ECG. This lack of data could have had an impact on the detection of ECG abnormalities. Additionally, we were not able to compare non-cDM1 and cDM1 in terms of cardiac features due to the small sample size of the non-congenital group. As a tertiary center, non-cDM1 patients are not systematically referred to our Institution and are frequently followed-up locally, which would explain the smaller proportion on non-cDM1 patients seen in our clinic. Moreover, no post-mortem examination was available for the four deceased children, which limits our ability to infer the prevalence of SCD in the group. We created our own systemic score considering the 8 most representative clinical features of cDM1 in our cohort. This could have resulted in the omission of other relevant clinical features not widely presented by our patients. Additionally, the power of the statistical tests is limited by the small number of patients included. Finally, no correlation could be made between cardiac involvement and CTG repeat length as repeat size is not routinely assessed by UK laboratories. This limits the cardiac phenotype-genotype correlation. Although genotype-phenotype correlations in terms of severity of cardiac involvement and size of the CTG expansion have been advocated in adult groups, this remains controversial. No targeted study in the cDM1 population, where the length of the repeat is maximal, has been carried out to date.

## Conclusion

This study demonstrates a high yield of cardiac conduction abnormalities with a progressive nature and potential for associated cardiac mortality due to dysrhythmias throughout childhood in an exclusively pediatric cDM1 cohort. Cardiac conduction disease is the most prevalent abnormality with 1st AVB demonstrated as the most frequent finding among cDM1 patients. Progression to higher degree of AVB was rare with only one patient requiring permanent pacing. There were four deaths despite the small cohort size, one due to progression of systemic disease but three were sudden and unexplained in otherwise stable children, and a sudden arrhythmic event could not be ruled out. Our findings stress the crucial role that regular and comprehensive cardiac follow-up of these patients plays starting from the onset of the disease. Further long-term prospective follow-up studies are needed to identify if electrocardiographic abnormalities can predict the risk of sudden cardiac events and namely sudden death. Genotype-phenotype correlations in terms of severity of cardiac involvement and size of the CTG expansion have been advocated in adult groups, this remains controversial. No targeted study in the cDM1 population, where the length of the repeat is maximal, has been carried out to date.

## Data Availability Statement

The original contributions presented in the study are included in the article/Supplementary Material, further inquiries can be directed to the corresponding author/s.

## Ethics Statement

The studies involving human participants were reviewed and approved by Research Board of Great Ormond Street Hospital. Written informed consent from the participants' legal guardian/next of kin was not required to participate in this study in accordance with the national legislation and the institutional requirements.

## Author Contributions

LB: drafted the manuscript, writing the bulk of the manuscript, and data collection. AH, JW, HW, and GN: data collection and critical revision of the manuscript. EF: data collection, statistical study, and critical revision of the manuscript. AM, FM, PM, SR, RQ, MS, GB, AS, LS, JK, and EC: critical revision of the manuscript. EC: coordinated and supervised data collection, editing, and responsible for the overall content. All authors have read and approved the final manuscript.

## Funding

This work was supported by the NIHR GOSH Biomedical Research Center. JK was supported by the British Heart Foundation (grant reference number: FS/16/72/32270), Medical Research Council Clinical Academic Partnership (MRC CARP, grant reference number: MR/T024062/1) award, Max's Foundation, Action Medical Research/LifeArc, and the Great Ormond Street Hospital Children's Charity. All other authors have reported that they have no relationships relevant to the contents of this paper to disclose.

## Conflict of Interest

The authors declare that the research was conducted in the absence of any commercial or financial relationships that could be construed as a potential conflict of interest.

## Publisher's Note

All claims expressed in this article are solely those of the authors and do not necessarily represent those of their affiliated organizations, or those of the publisher, the editors and the reviewers. Any product that may be evaluated in this article, or claim that may be made by its manufacturer, is not guaranteed or endorsed by the publisher.

## References

[1] Wahbi K Babuty D Probst V Wissocque L Labombarda F Porcher R. Incidence and predictors of sudden death, major conduction defects and sustained ventricular tachyarrhythmias in 1388 patients with myotonic dystrophy type 1. Eur Heart J. (2016) 38:751–8. 10.1093/eurheartj/ehw569 27941019

[2] Šabovič M Medica I Logar N Mandić E Zidar J Peterlin B. Relation of CTG expansion and clinical variables to electrocardiogram conduction abnormalities and sudden death in patients with myotonic dystrophy. Neuromuscul Disord. (2003) 13:822–6. 10.1016/S0960-8966(03)00138-X 14678805

[3] Lau JK Sy RW Corbett A Kritharides L. Myotonic dystrophy and the heart: a systematic review of evaluation and management. Int J Cardiol. (2015) 184:600–8. 10.1016/j.ijcard.2015.03.069 25769007

[4] Dogan C De Antonio M Hamroun D Varet H Fabbro M Rougier F. Gender as a modifying factor influencing myotonic dystrophy type 1 phenotype severity and mortality: a nationwide multiple databases cross-sectional observational study. PLoS One. (2016) 11:1–12. 10.1371/journal.pone.0148264 26849574 PMC4744025

[5] Pelargonio G Russo A. Dello Sanna T De Martino G Bellocci F. Myotonic dystrophy and the heart. Heart. (2002) 88:665–70. 10.1136/heart.88.6.665 12433913 PMC1767476

[6] Ho G Cardamone M Farrar M. Congenital and childhood myotonic dystrophy: Current aspects of disease and future directions. World J Clin Pediatr. (2015) 4:66–80. 10.5409/wjcp.v4.i4.66 26566479 PMC4637811

[7] Ho G Carey KA Cardamone M Farrar MA. Myotonic dystrophy type 1: clinical manifestations in children and adolescents. Arch Dis Child. (2019) 104:48–52. 10.1136/archdischild-2018-314837 29871899

[8] Feingold B Mahle WT Auerbach S Clemens P Domenighetti AA Jefferies JL. Management of cardiac involvement associated with neuromuscular diseases: a scientific statement from the American Heart Association. Circulation. (2017) 136:200–31. 10.1161/CIR.0000000000000526 28838934

[9] Bassez G Lazarus A Desguerre I Varin J Laforêt P Bécane HM. Severe cardiac arrhythmias in young patients with myotonic dystrophy type 1. Neurology. (2004) 63:1939–41. 10.1212/01.WNL.0000144343.91136.CF 15557517

[10] European Society of Cardiology (ESC) European Heart Rhythm Association (EHRA) Brignole M Auricchio A Baron-Esquivias G Bordachar P. 2013 ESC guidelines on cardiac pacing and cardiac resynchronization therapy: the task force on cardiac pacing and resynchronization therapy of the European Society of Cardiology (ESC). Developed in collaboration with the European Heart Rhythm Association . Europace. (2013) 15:1070–118. 10.1093/europace/eut206 23801827

[11] Priori SG Blomstrom-Lundqvist C Mazzanti A Blom N Borggrefe M Camm J. 2015 ESC Guidelines for the management of patients with ventricular arrhythmias and the prevention of sudden cardiac death. Eur Heart J. (2015) 36:2793–867. 10.1093/eurheartj/ehv316 26745817

[12] Rijnbeek PR Van Herpen G Bots ML Man S Verweij N Hofman A. Normal values of the electrocardiogram for ages 16-90 years. J Electrocardiol. (2014) 47:914–21. 10.1016/j.jelectrocard.2014.07.022 25194872

[13] Rijnbeek PR Witsenburg M Schrama E Hess J Kors JA. New normal limits for the paediatric electrocardiogram. Eur Heart J. (2001) 22:702–11. 10.1053/euhj.2000.2399 11286528

[14] Surawicz B Childers R Deal BJ Gettes LS. AHA/ACCF/HRS recommendations for the standardization and interpretation of the electrocardiogram. Part III: Intraventricular conduction disturbances: a scientific statement from the American Heart Association Electrocardiography and Arrhythmias Committee. Circulation. (2009) 119:235–40. 10.1161/CIRCULATIONAHA.108.191095 19228822

[15] DePace NL Colby J Hakki AH Manno B Horowitz LN Iskandrian AS. Poor R wave progression in the precordial leads: Clinical implications for the diagnosis of myocardial infarction. J Am Coll Cardiol. (1983) 2:1073–9. 10.1016/S0735-1097(83)80332-5 6630780

[16] Mussinelli R Salinaro F Alogna A Boldrini M Raimondi A Musca F. Diagnostic and prognostic value of low QRS voltages in cardiac AL amyloidosis. Ann Noninvasive Electrocardiol. (2013) 18:271–80. 10.1111/anec.12036 23714086 PMC6932192

[17] Marcus FI McKenna WJ Sherrill D Basso C Bauce B Bluemke DA et al. Diagnosis of arrhythmogenic right ventricular cardiomyopathy/dysplasia: proposed modification of the task force criteria. Circulation. (2010) 121:1533–41. 10.1161/CIRCULATIONAHA.108.840827 20172911 PMC2860804

[18] Rautaharju PM Surawicz B Gettes LS. AHA/ACCF/HRS Recommendations for the Standardization and Interpretation of the Electrocardiogram. Part IV: The ST Segment, T and U Waves, and the QT Interval A Scientific Statement From the American Heart Association Electrocardiography and Arrhythmias. J Am Coll Cardiol. (2009) 53:982–91. 10.1016/j.jacc.2008.12.014 19281931

[19] Lopez L Colan S Stylianou M Granger S Trachtenberg F Frommelt P. Relationship of echocardiographic Z Scores adjusted for body surface area to age, sex, race, and ethnicity: the pediatric Heart Network Normal Echocardiogram Database. Circ Cardiovasc Imaging. (2017) 10:1–7. 10.1161/CIRCIMAGING.117.006979 29138232 PMC5812349

[20] Evangelista A Flachskampf F Lancellotti P Badano L Aguilar R Monaghan M. European Association of Echocardiography recommendations for standardization of performance, digital storage and reporting of echocardiographic studies. Eur J Echocardiogr. (2008) 9:438–48. 10.1093/ejechocard/jen174 18579482

[21] Petri H Vissing J Witting N Bundgaard H Kober L. Cardiac manifestations of myotonic dystrophy type 1. Int J Cardiol. (2012) 160:82–8. 10.1016/j.ijcard.2011.08.037 21917328

[22] Sharma A Singh S Mishra SK. Cardiac abnormalities in congenital and childhood Myotonic muscular dystrophy type 1. Neuropediatrics. (2017) 48:42–4. 10.1055/s-0036-1597546 27992942

[23] Choudhary P Nandakumar R Greig H Broadhurst P Dean J Puranik R. Structural and electrical cardiac abnormalities are prevalent in asymptomatic adults with myotonic dystrophy. Heart. (2016) 102:1472–8. 10.1136/heartjnl-2015-308517 27164920

[24] Usoro AO Bradford N Shah AJ Soliman EZ. Risk of mortality in individuals with low QRS voltage and free of cardiovascular disease. Am J Cardiol. (2014) 113:1514–7. 10.1016/j.amjcard.2014.02.006 24630386

