# Supplementary material for: Cardiac Manifestations of Myotonic Dystrophy in a Pediatric Cohort
Source: Front Pediatr. 2022 Jun 9;10:910660. doi: 10.3389/fped.2022.910660 (PMC9218560; doi:10.3389/fped.2022.910660)

Supplementary figure 1. Column chart showing the number of extra-cardiac variants affected in congenital DM1 pediatric patients.

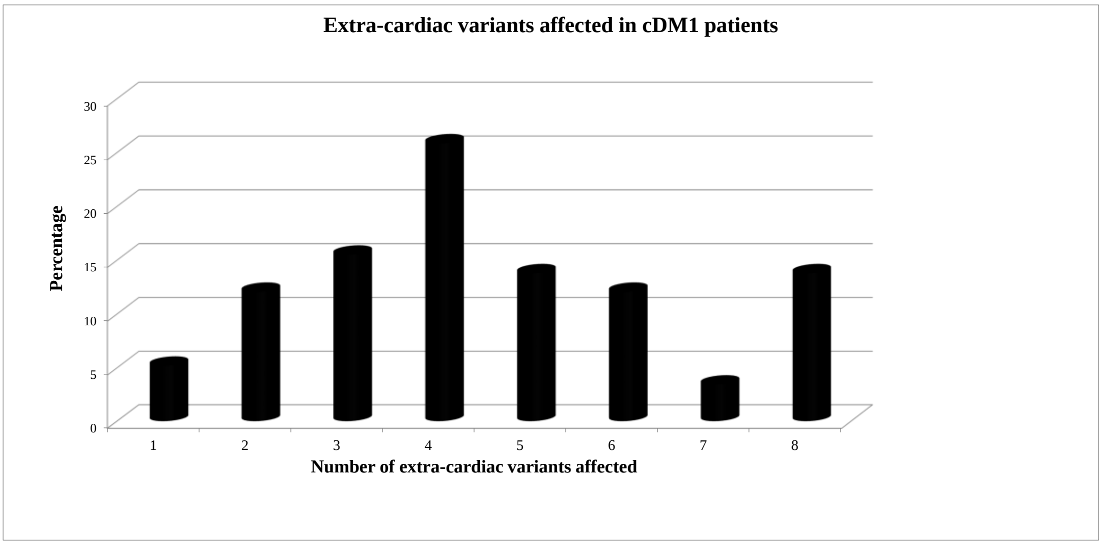

Supplement: Supplementary file 1 [file Image_1.pdf]
