# Supplementary material for: Cardiac Manifestations of Myotonic Dystrophy in a Pediatric Cohort
Source: Front Pediatr. 2022 Jun 9;10:910660. doi: 10.3389/fped.2022.910660 (PMC9218560; doi:10.3389/fped.2022.910660)
Supplement: Supplementary file 2 [file Image_2.pdf]

Supplementary figure 2. Diagnostic work-up in congenital DM1 pediatric cohort.

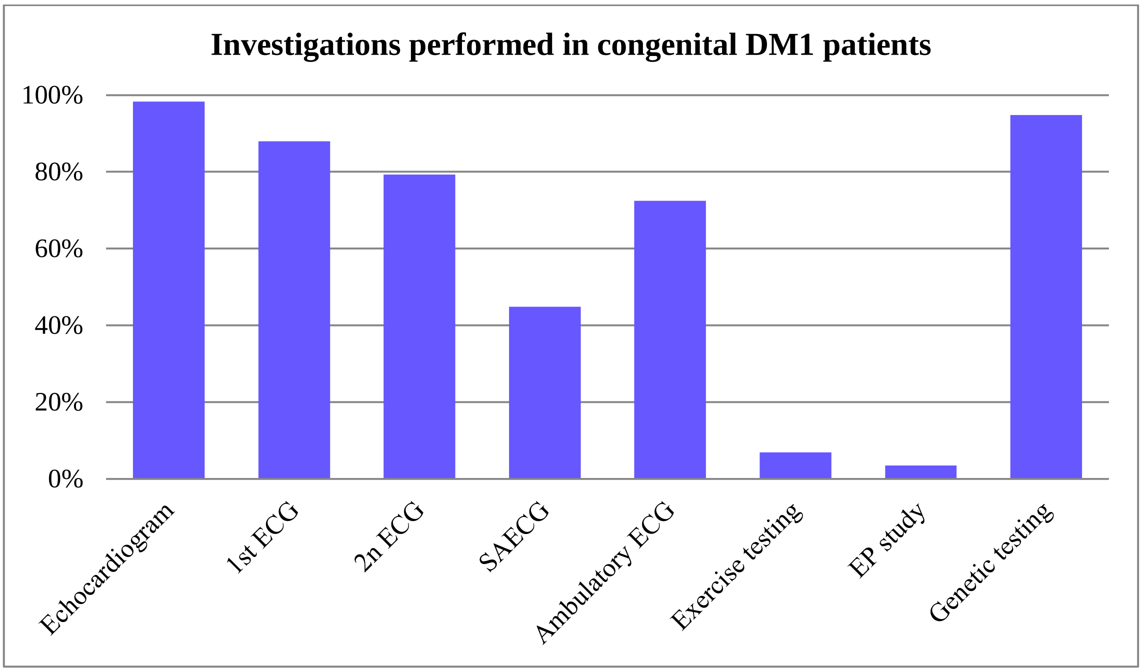

1st: first; 2nd: second; ECG: electrocardiogram; EP: electrophysiological; SAECG: signal averaged ECG.
