# Supplementary material for: Cardiac Manifestations of Myotonic Dystrophy in a Pediatric Cohort
Source: Front Pediatr. 2022 Jun 9;10:910660. doi: 10.3389/fped.2022.910660 (PMC9218560; doi:10.3389/fped.2022.910660)

**Suplementary figure 3.** QRS and PR interval duration at baseline and follow-up 12-lead ECG, separated for gender and in relation to the 98th and 2nd percentiles.

A. QRS duration at baseline and follow-up 12-lead ECG, separated for gender and in relation to the 98th and 2nd percentiles.

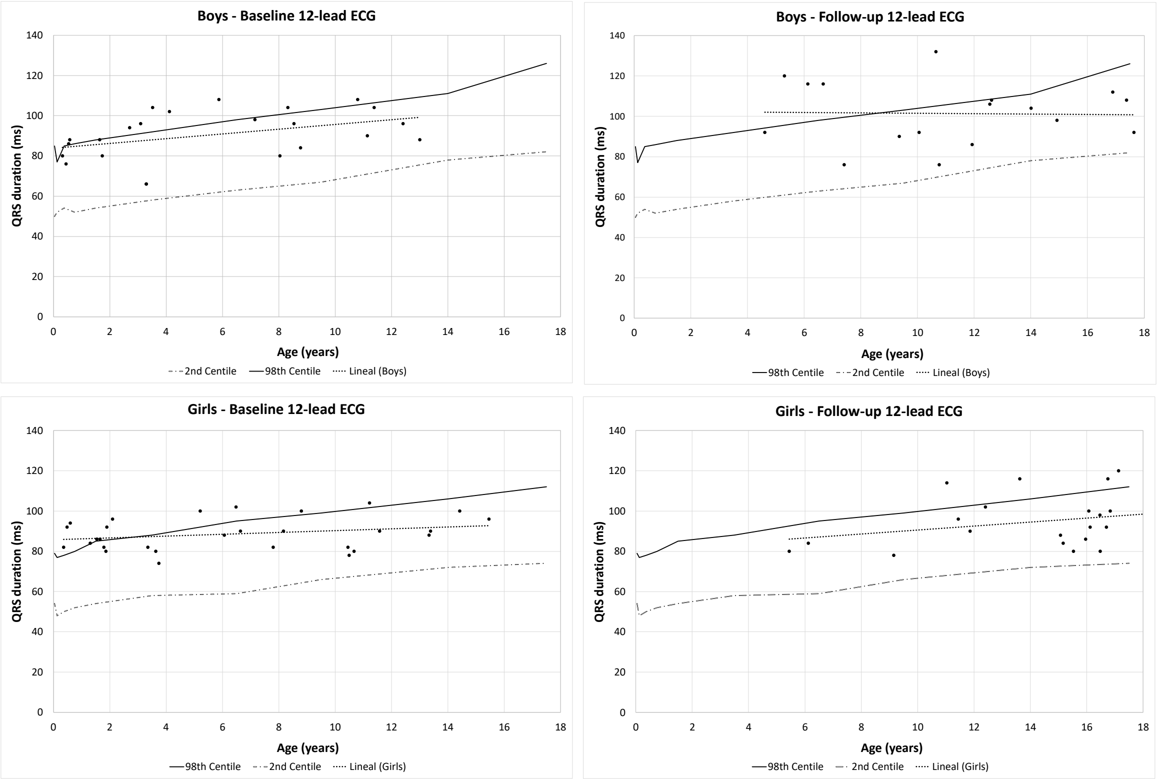


B. PR interval duration at baseline and follow-up 12-lead ECG, separated for gender and in relation to the 98th and 2nd percentiles.

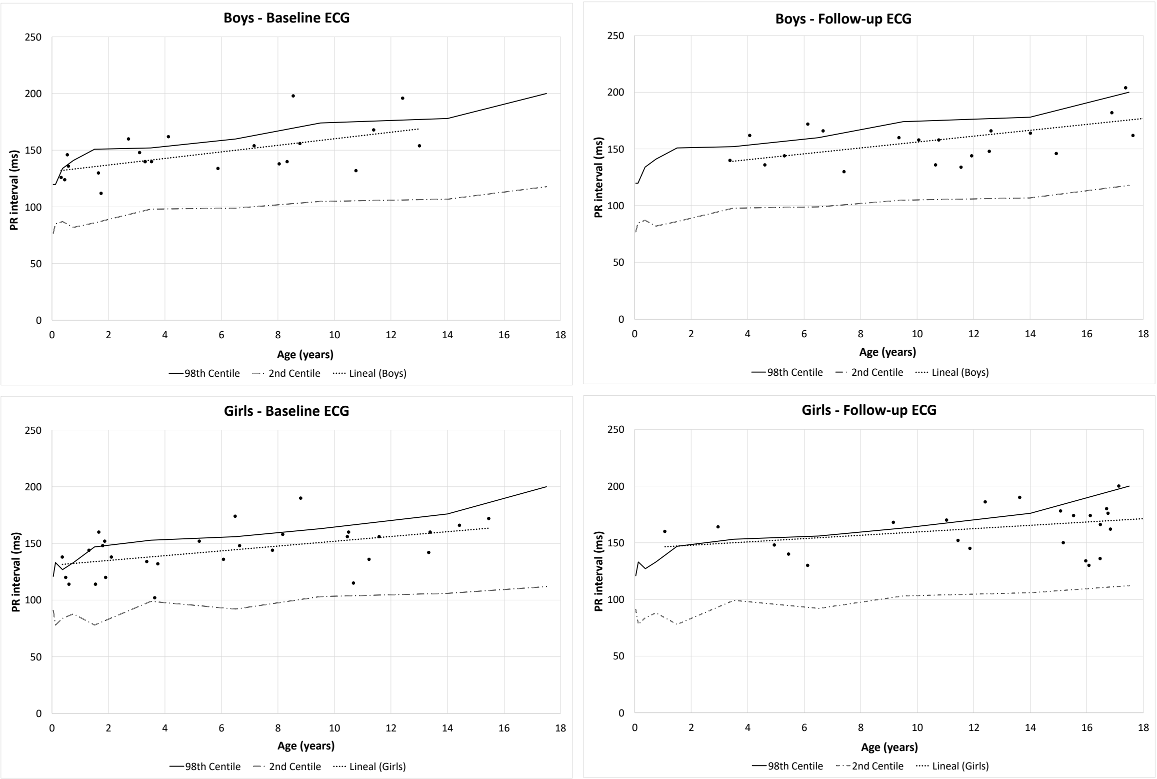

Supplement: Supplementary file 3 [file Image_3.pdf]
