# Supplementary material for: Cardiac Manifestations of Myotonic Dystrophy in a Pediatric Cohort
Source: Front Pediatr. 2022 Jun 9;10:910660. doi: 10.3389/fped.2022.910660 (PMC9218560; doi:10.3389/fped.2022.910660)
Supplement: Supplementary file 4 [file Table_1.pdf]

Supplementary table 1. Extracardiac features in DM1 paediatric cohort.

	**cDM1 (n=56)**	**iDM1 and jDM1 (n=11)**
Neuromuscular		
Delayed milestones	55 (98.2)	1 (9.1)
**Learning difficulties**	56 (100.0)	6 (54.5)
Speech and language delay	52 (92.9)	5 (45.5)
**Full wheelchair dependence**	4 (7.1)	0 (0.0)
**Partial wheelchair dependence**	43 (76.8)	0 (0.0)
Respiratory		
**Non-invasive ventilation**	21 (37.5)	2 (18.2)
Recurrent chest infections	15 (26.8)	0 (0.0)
**Sleep disordered breathing**	23/54 (42.6)	1 (9.1)
Gastrointestinal		
**Faecal incontinence**	34/52 (65.4)	2 (18.2)
Constipation	23 (41.1)	2 (18.2)
**NG feeding/Gastrostomy feeding**	21 (37.5)	0 (0.0)
**Dysphagia**	19 (33.9)	0 (0.0)
Liver transplant for cirrhosis	1 (1.8)	0 (0.0)
**Urinary incontinence**	35/50 (70.0)	2 (18.2)
Cataracts	1 (1.8)	1 (9.1)

Hearing loss	2 (3.6)	0 (0.0)

Fractions give the absolute number of patients divided by the number of patients with available clinical information for each item. Values are n (%). In bold and italics features used to define clinical severity.

cDM1: Congenital Myotonic Dystrophy type 1; FH: Family History; iDM1: Infantile Myotonic Dystrophy type 1; jDM1: Juvenile Myotonic Dystrophy type 1; NG: Nasogastric.
