# Supplementary material for: Cardiac Manifestations of Myotonic Dystrophy in a Pediatric Cohort
Source: Front Pediatr. 2022 Jun 9;10:910660. doi: 10.3389/fped.2022.910660 (PMC9218560; doi:10.3389/fped.2022.910660)
Supplement: Supplementary file 5 [file Table_2.docx]

**Supplementary table 2.** Comparison of ECG characteristics in the deceased and alive paediatric congenital DM1 patients (with at least one available ECG).

| **Baseline information** | **cDM1 deceased (n=3)*,** n (%) | **cDM1 alive (n=49),** n (%) | **P-value** |
| --- | --- | --- | --- |
| Conduction defect | 1 (33.3) | 22 (44.9) | 0.636 |
| 1^st^ AVB | 1 (33.3) | 20 (40.8) | >0.999 |
| QRS axis deviation | 3 (100.0) | 36 (73.5) | 0.587 |
| LAFB | 0 (0.0) | 5 (10.2) | >0.999 |
| RBBB | 0 (0.0) | 2 (4.1) | >0.999 |
| Low QRS voltages | 3 (100.0) | 15 (30.6) | 0.093 |
| Abnormal repolarisation | 3 (100.0) | 17 (34.7) | 0.138 |
| Poor R wave progression | 0 (0.0) | 9 (18.4) | >0.999 |
| Intravent conduction delay | 1 (33.3) | 15 (30.6) | >0.999 |

AVB: atrioventricular block; cDM1: congenital myotonic dystrophy type 1; intravent: intraventricular; LAFB: left anterior fascicular block; SAECG: signal averaged ECG.

*4 patients died during follow-up but the ECG was only available for 3 of them.
