# Supplementary material for: Cardiac Manifestations of Myotonic Dystrophy in a Pediatric Cohort
Source: Front Pediatr. 2022 Jun 9;10:910660. doi: 10.3389/fped.2022.910660 (PMC9218560; doi:10.3389/fped.2022.910660)
Supplement: Supplementary file 6 [file Table_3.docx]

**Supplementary table 3.** Statistical association between mortality during follow-up and presence of systemic features of paediatric congenital DM1 patients.

| **Systemic feature** | **P-value** |
| --- | --- |
| Non-invasive ventilation | 0.143 |
| Faecal incontinence | 0.285 |
| Nasogastric feeding/gastrostomy | 0.287 |
| Dysphagia | 0.108 |
| Sleep disorders | 0.301 |
| Urinary incontinence | > 0.999 |
| Wheelchair dependence | > 0.999 |
