# Supplementary material for: Cardiac Manifestations of Myotonic Dystrophy in a Pediatric Cohort
Source: Front Pediatr. 2022 Jun 9;10:910660. doi: 10.3389/fped.2022.910660 (PMC9218560; doi:10.3389/fped.2022.910660)
Supplement: Supplementary file 7 [file Table_4.docx]

**Supplementary table 4.** Signal-averaged ECG (SAECG) findings of paediatric congenital DM1 patients.

| **SAECG** | **Study population (n=26)** |
| --- | --- |
| Negative for late potentials, n (%) | 9 (34.6%) |
| Positive in 2 of 3 parameters, n (%) | 13 (50%) |
| Positive in all 3 parameters, n (%) | 10 (38.5%) |
| Abnormal increase of filtered QRS duration, n (%) – years [IQR] | 4 (15.4%) - 10.9 [5.3 – 12.0] |
| Positive for root-mean-square voltage of terminal 40ms and low-amplitude signal duration of terminal QRS <40ms, n (%) – years [IQR] | 3 (11.5%) - 11.2 [5.3 – 12.4] |
