# Supplementary material for: Cardiac Manifestations of Myotonic Dystrophy in a Pediatric Cohort
Source: Front Pediatr. 2022 Jun 9;10:910660. doi: 10.3389/fped.2022.910660 (PMC9218560; doi:10.3389/fped.2022.910660)
Supplement: Supplementary file 9 [file Table_6.docx]

**Supplementary table 6.** Statistical differences in median systemic severity score between patients with and without ECG abnormalities.

| **ECG abnormality** | **P-value** |
| --- | --- |
| 1^st^ AVB | 0.226 |
| QRS axis deviation | 0.237 |
| LAFB | 0.995 |
| RBBB | 0.866 |
| Low QRS voltages | 0.063 |
| Abnormal repolarisation | 0.782 |
| Poor R wave progression | 0.678 |
| Intraventricular conduction delay | 0.198 |

AVB: atrioventricular block; LAFB: left anterior fascicular block.
